# Supplementary figures and images for: Single-Fluorescent Protein Reporters Allow Parallel Quantification of Natural Killer Cell-Mediated Granzyme and Caspase Activities in Single Target Cells
Source: Front Immunol. 2018 Aug 8;9:1840. doi: 10.3389/fimmu.2018.01840 (PMC6092488; doi:10.3389/fimmu.2018.01840)

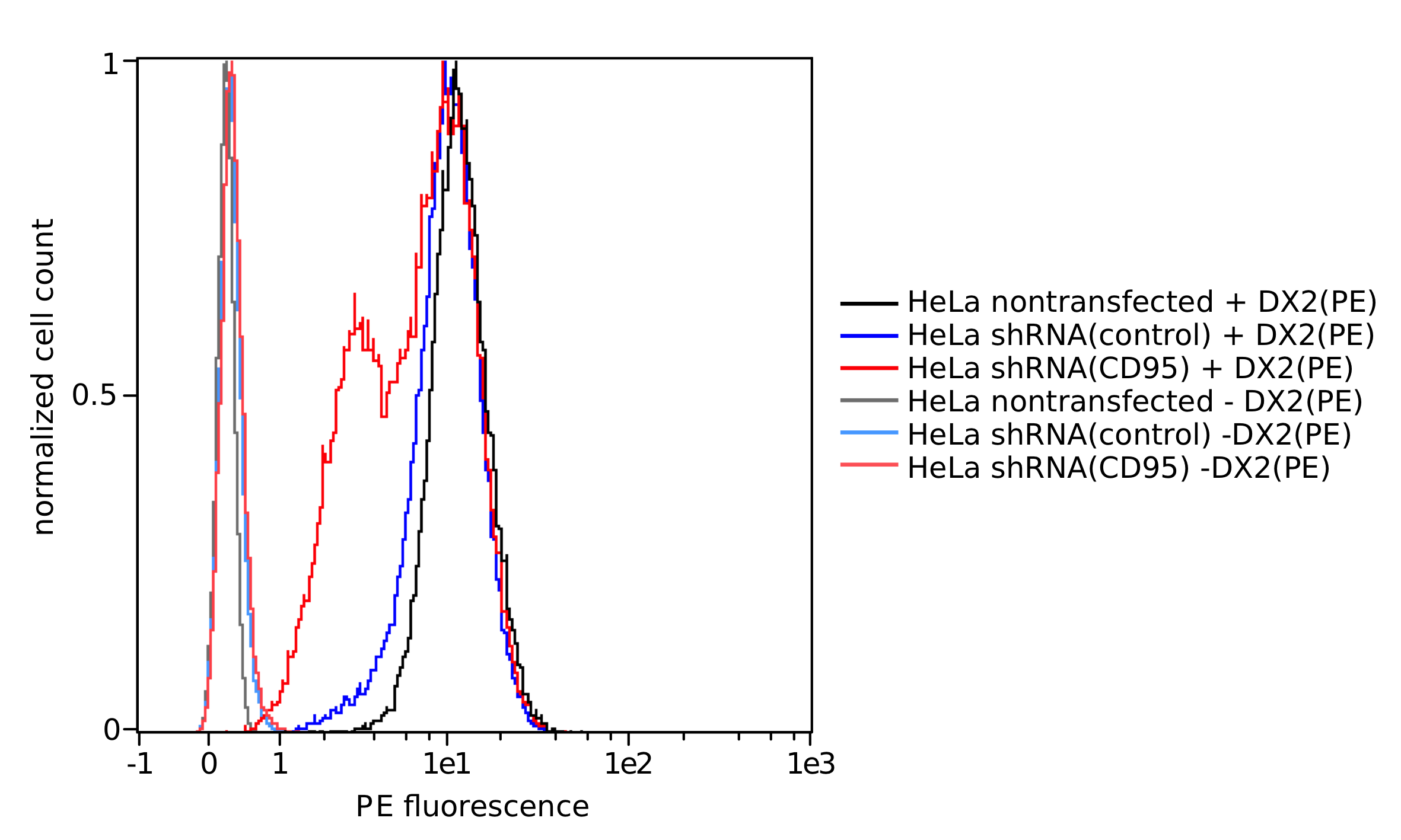

Supplement: Figure S1 — Transient knockdown of CD95 in HeLa cells. HeLa cells were non transfected (black line) or transfected with a scrambled shRNA (blue line) or a shRNA against CD95 (red line). Cells were stained 3 days after transfection with or without the anti-CD95 antibody DX2. [file image_1.tif]

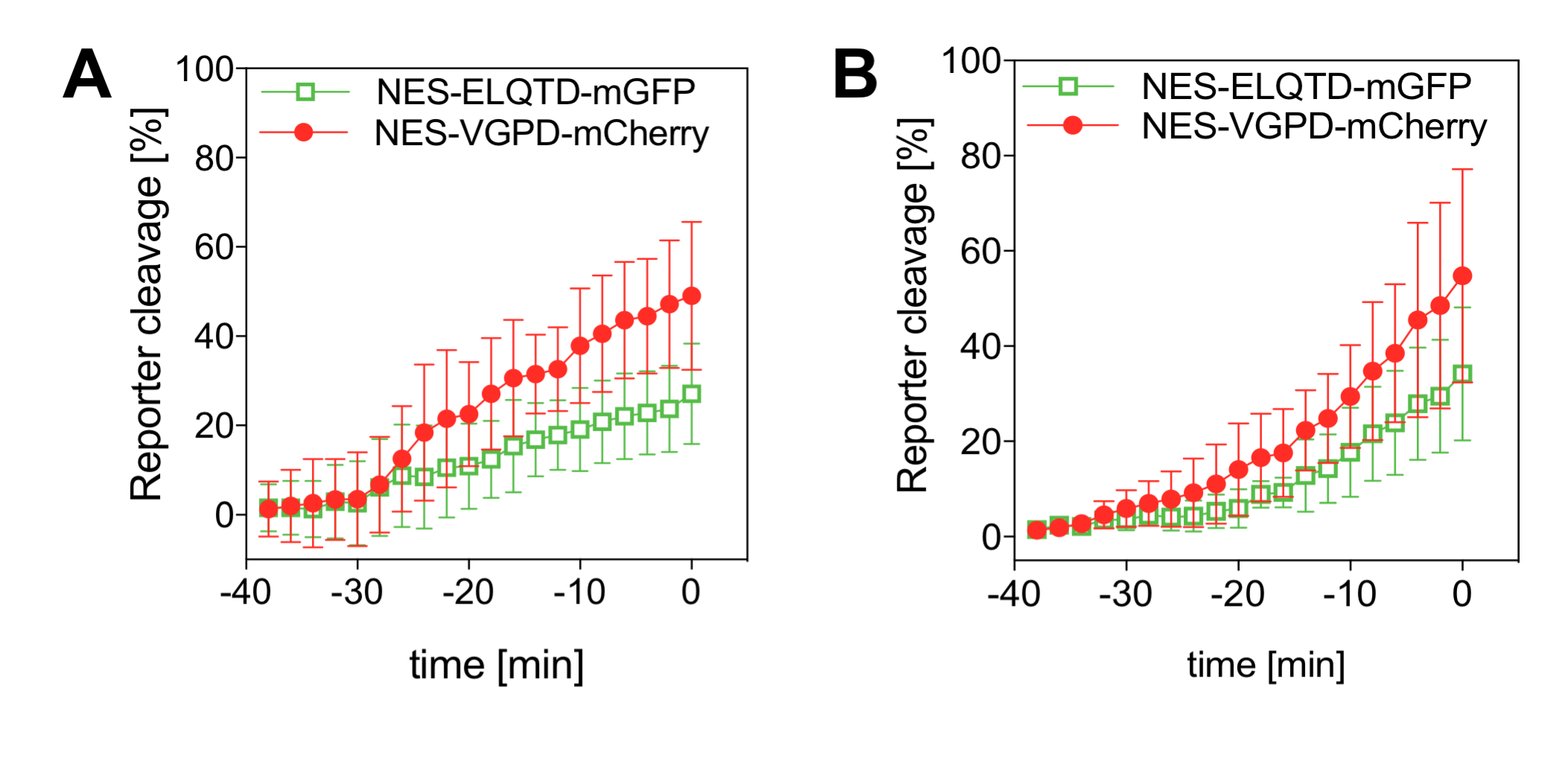

Supplement: Figure S2 — Granzyme B and caspase-8 activity in HeLa and MDA-MB-468 cells upon killing by primary natural killer (NK) cells. (A) HeLa-CD48 and (B) MDA-MB-468 cells were transfected with NES-ELQTD-mGFP (for caspase-8) and NES-VGPD-mCherry for granzyme B and incubated with activated primary human NK cells. Reporter cleavage was analyzed as described in Figure 4. [file image_2.tif]
